# A rare case of ectopic ACTH syndrome originating from malignant renal paraganglioma

**DOI:** 10.1590/2359-3997000000240

**Published:** 2017-01-27

**Authors:** Esra Tutal, Demet Yılmazer, Taner Demirci, Evrim Cakır, Salih Sinan Gültekin, Bahadır Celep, Oya Topaloğlu, Erman Çakal

**Affiliations:** 1 Diskapi Yildirim Beyazit Teaching and Research Hospital Department of Endocrinology and Metabolism Irfan Bastug Caddesi Ankara Turkey Diskapi Yildirim Beyazit Teaching and Research Hospital, Department of Endocrinology and Metabolism, Irfan Bastug Caddesi, Ankara, Turkey; 2 Department of Endocrinology and Metabolism Diskapi Teaching and Research Hospital Irfan Bastug Caddesi Ankara Turkey Department of Endocrinology and Metabolism, Diskapi Teaching and Research Hospital, Irfan Bastug Caddesi, Ankara, Turkey; 3 Department of Nuclear Medicine Diskapi Teaching and Research Hospital Irfan Bastug Caddesi Ankara Turkey Department of Nuclear Medicine, Diskapi Teaching and Research Hospital, Irfan Bastug Caddesi, Ankara, Turkey; 4 Department of General Surgery Diskapi Teaching and Research Hospital Irfan Bastug Caddesi Ankara Turkey Department of General Surgery, Diskapi Teaching and Research Hospital, Irfan Bastug Caddesi, Ankara, Turkey

## Abstract

Ectopic adrenocorticotropic hormone (ACTH) syndrome is characterized by hypercortisolism due to the hypersecretion of a non-pituitary ACTH-secreting tumor leading to Cushing’s syndrome. Only a few cases have been reported previously as causing ectopic ACTH related to paraganglioma. Herein, we present a case of Cushing’s syndrome, in who was proved to be attributable to an ACTH-secreting renal malignant paraganglioma. A 40-year-old woman presented with a five-month history of newly diagnosed hypertension and diabetes, weakness, hyperpigmentation, oligomenorrhea, hirsutism, and acneiform lesions. She showed cushingoid features, including moon face, facial hirsutism, facial and truncal acne, hyperpigmentation, and severe muscle weakness of the limbs. She did not have other findings such as striae, supraclavicular fat accumulation, and buffalo hump. Laboratory examination showed the presence of hypopotasemia, hyperglycemia, hyperthyroidism, and leukocytosis. The serum levels of ACTH, cortisol, and urine-free cortisol were markedly elevated. Results of an overnight 2-mg dexamethasone suppression test included a basal serum cortisol of 61.1 mcg/dL (normal range: 4.6-22.8 mcg/dL) and a cortisol value of 46.1 mcg/dL after dexamethasone administration. There was no suppression found after 2-day 8-mg dexamethasone administration. Magnetic resonance imaging (MRI) of the pituitary gland indicated two microadenomas. An abdominal MRI scan revealed horseshoe kidney, bilateral adrenal hyperplasia, and masses with dimensions of 35 x 31 mm in the left kidney. Inferior petrosal sinus sampling showed no evidence of a central-to-peripheral gradient of ACTH. A positron emission tomography/computed tomography scan showed intense increased activity in the lower pole of the left kidney. Left adrenalectomy and left partial nephrectomy were performed. The resected tumor was diagnosed as the ACTH-secreting paraganglioma in the pathological examination, which was confirmed by immunohistochemical studies with chromogranin A, synaptophysin, and ACTH. Only a few cases of paragangliomas as a cause of ectopic ACTH syndrome have been reported. To our knowledge, this is the first case of renal paraganglioma resulting in Cushing’s syndrome due to ectopic ACTH hypersecretion.

## INTRODUCTION

Ectopic adrenocorticotropic hormone (ACTH) syndrome is characterized by hypercortisolism with bilateral adrenocortical hyperplasia and hyperfunction due to the hypersecretion of non-pituitary ACTH-secreting tumor, which leads to Cushing’s syndrome. Ectopic ACTH syndrome appears in approximately 10-15% of adult patients with Cushing’s syndrome ( 1 ). Most cases of ectopic ACTH syndrome are caused by malignancies, including the small-cell type of lung carcinomas, thymic carcinoids, islet cell tumors of the pancreas, medullary carcinoma of the thyroid, and bronchial adenomas or carcinoids. Paragangliomas are rare tumors that arise from neural crest cells and are associated with autonomic ganglia. Pheochromocytomas that cause ectopic ACTH syndrome are very rare ( 2 ). A few cases with ACTH-secreting paragangliomas have been previously reported, which have been localized in the paranasal sinus ( 3 , 4 ), cervical ( 5 ), mediastinal/thoracic ( 6 - 8 ), and retroperitoneal ( 9 ) regions. To the best of our knowledge, there hasn’t been any published report in the literature about ACTH-secreting renal malignant paraganglioma.

In this report, we present a case of a 40-year-old woman diagnosed with Cushing’s syndrome, which proved to be attributable to an ACTH-secreting renal malignant paraganglioma.

## CASE REPORT

A 40-year-old woman presented with a five-month history of newly diagnosed hypertension and diabetes, weakness, hyperpigmentation, oligomenorrhea, hirsutism, and acneiform lesions. Physical examination revealed a blood pressure of 140/95 mmHg, a heart rate of 82 beats/min, weight of 58 kg, and height of 155 cm. She showed cushingoid features including moon face, facial hirsutism, facial and truncal acne, hyperpigmentation, and severe muscle weakness of the limbs. She did not show findings such as striae, supraclavicular fat accumulation, and buffalo hump. The thyroid examination revealed a 2 cm diameter nodule. Her mood was not depressed. She did not give a special medical problem history in her family. Laboratory examination showed the presence of hypopotasemia, hyperglycemia, hyperthyroidism, and leukocytosis ( Table 1 ). The serum levels of ACTH, cortisol, and urine-free cortisol were markedly elevated ( Table 2 ). Results of an overnight 2-mg dexamethasone suppression test included a basal serum cortisol of 61.1 mcg/dL (normal range: 4.6-22.8 mcg/dL) and a cortisol value of 46.1 mcg/dL after the dexamethasone administration. There was no suppression after 2-day 8-mg dexamethasone administration ( Table 3 ). We didn’t find any elevation of urinary metanephrine and nometanephrine levels. The patient gave her written informed consent.

**Table 1:** Baseline laboratory values of the patient

	Results	Normal range
WBC	11.17	5.2-11.4 10^3/µL
Hg	11.7	12-18 g/dL
Plt	218	130-400 10^3/µL
Neutrophil	9.45	1.9-8 10^3/µL
Eosinophil	0	0-0.8 10^3/µL
Lymphocyte	1.11	0.9-5.2 10^3/µL
Glucose	133	70-100 mg/dL
Na	143	136-145 mmol/L
K	2.8	3.5-5.1 mmol/L

WBC: white blood cell; Hg: hemoglobin; Plt: platelet; Na: sodium; K: potassium.

**Table 2:** Baseline hormonal values of the patient

	Results	Normal range
ACTH (basal)	679	0-46 pg/mL
Cortisol (basal)	61.1	4.6-2.28 ug/dL
Urinary free cortisol	2032.5	3.5-5.5 ug/24 hr
Free T4	1.14	0.74-1.52 ng/dL
Free T3	2.7	2.3-4.2 pg/mL
TSH	0.07	0.64-6.27 muI/L
FSH	4.9	IU/L
LH	1.0	IU/L
Estradiol	57.8	pg/mL
PRL	4.4	3.4-29.8 ng/mL
GH	1.05	0-10 ng/mL
Total testosterone	98.45	14.2-73.1 ng/dL

ACTH: adrenocorticotropic hormone; TSH: thyroid-stimulating hormone; FSH: follicle-stimulating hormone; LH: luteinizing hormone; PRL: prolactin; GH: growth hormone.

**Table 3:** Results of the 2-mg and 8-mg dexametasone supression tests

	ACTH (pg/mL)	Cortisol (mcg/dL)	Urinary free cortisol (ug/d)
Basal	679	61.1	2032, 1850*
2 mg DST		46.1	
8 mg DST	856	46.8	1030

* Urinary free cortisol were measured two times.

**Figure 1:**
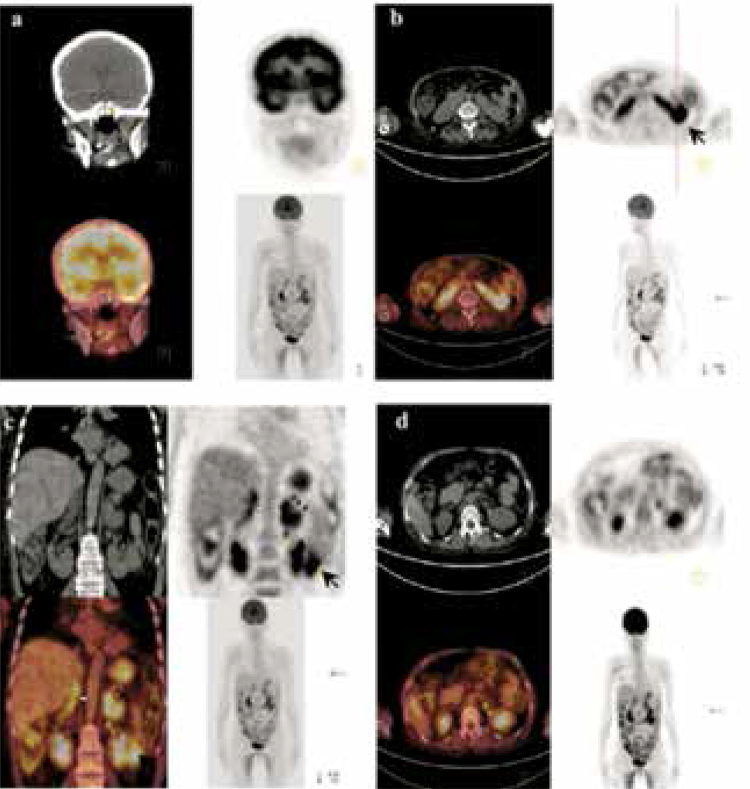
PET/CT scan. (A) Shows a mild focal uptake in the pituitary gland region. (B) Demonstrates horseshoe kidney deformity and intense increased activity in the lower pole of the left kidney (thick black arrow). (C, D) Show heterogeneous increased uptake in the right adrenal gland (thick white arrow), intense increased uptake in the left adrenal gland (black arrowheads), and a focal increased uptake in a lymph node located in the inferior adjacent to the left adrenal gland (thin yellow arrows).

Thyroid scintigraphy showed a hyperactive nodule, which was localized in the right lobe of the thyroid gland ( Figure 1 ).

**Figure 2:**
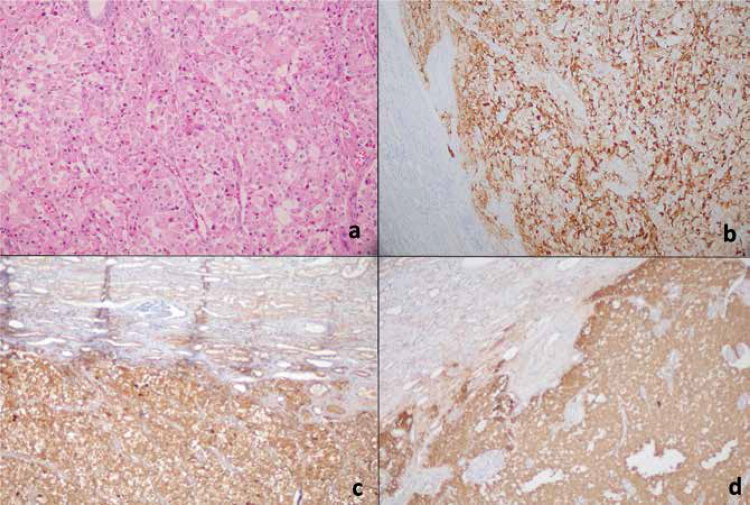
Hematoxylin and eosin (H&E) stain of the tumor in the kidney (A), immunohistochemistry with antibodies specific for ACTH (B), chromogranin A (C), and synaptophysin (D).

Magnetic resonance imaging (MRI) of the pituitary gland indicated two microadenomas at the mid-anterior and left-posterior sites. An abdominal MRI scan revealed horseshoe kidney, bilateral adrenal hyperplasia, and masses with dimensions of 35 x 31 mm in the left kidney ( Figure 2 ). Thoracic MRI findings were normal.

We did not found any increment after desmopressin injection. Inferior petrosal sinus sampling showed that there was no evidence of a central-to-peripheral gradient of ACTH at baseline or after administration of ovine corticotropin-releasing hormone (CRH) ( Table 4 ).

**Table 4:** Results of Inferior Petrosal Sinus Sampling at baseline and after administration of ovine CRH

	ACTH (right)	ACTH (left )	ACTH (peripheral)
-2 min	848	832	812
0 min	866	904	814
2 min	916	948	846
5 min	858	818	795
10 min	866	837	839

A positron emission tomography/computed tomography (PET/CT) scan was performed using ^18^F-fluorodeoxyglucose (FDG). The images ( Figure 1 ) showed several pathological uptakes. A mild focal uptake with the maximum standardized uptake value (SUV_max_: 2.76) was observed in the pituitary gland region. The horseshoe kidney deformity, in addition to an intense increased activity in the lower pole of the left kidney (SUV_max_: 9.87), was detected. There was a bilateral increased uptake in both adrenal glands with the right one having a heterogeneous character (left SUV_max_: 7.21 and right SUV_max_: 6.21). A focal increased uptake (SUV_max_: 4.31) in a lymph node located in the inferior adjacent to the left adrenal gland was observed.

**Figure 3:**
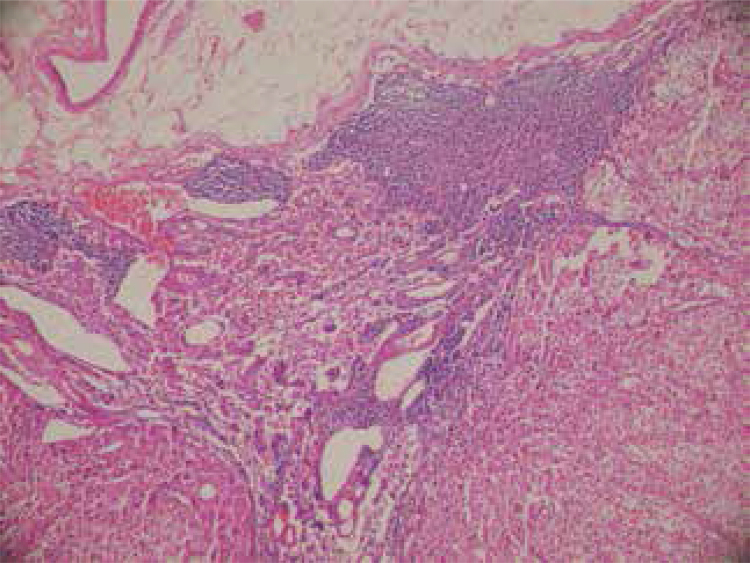
Hematoxylin and eosin (H&E) stain of the metastatic periadrenal lymph node.

Left adrenalectomy and left partial nephrectomy were performed. The resected tumor was diagnosed as the ACTH-secreting paraganglioma in the pathological examination, which was confirmed by immunohistochemical studies with chromogranin A, synaptophysin, and ACTH ( Figure 2 ). The periadrenal lymph node was evaluated as a metastatic lymph node ( Figure 3 ). The left adrenal mass was assessed as compatible with the adrenal hyperplasia. After surgical resection of the paraganglioma, the patient’s blood glucose and potassium levels have gradually returned to the near normal ranges without medication. Postoperative levels of plasma ACTH and cortisol returned to the normal ranges. She needed a 7.5-mg dose of prednisolone per day. In addition, one month after surgery, the patient was treated with radioiodine ^131^I in a dose of 740 MBq (20 mCi) due to clinically apparent hyperthyroidism.

## DISCUSSION

Paragangliomas are rare tumors arising from chromaffin tissue cells derived from the embryonic neural crest. Paragangliomas may be located between the cervical region and the lower pelvis cavity (wherever sympathetic or parasympathetic ganglia are present). Most of these tumors arise sporadically in the later period of life, especially after the sixth decade. Approximately 20% of these tumors are malignant ( 10 ).

In the majority of cases, paragangliomas of the head and neck are benign. Typically, these tumors are asymptomatic, but sometimes, catecholamine excess symptoms and signs, including hypertension, diabetes, and hypermetabolism, may be seen. Hypersecretion of catecholamines were not found in our patient. However, 15-35% of abdominal paragangliomas are malignant, especially in patients who have mutations of the gene encoding the B subunit of the mitochondrial complex II enzyme succinate dehidrogenase enzyme subunit B (SDHB) ( 11 ). We could not evaluate SDHB mutation in our patient for financial reasons. Malignancy is defined by the presence of metastases, and the most common sites for metastases of malignant paragangliomas are lymphatic nodes (local or distant), as in our patient. The other common sites for metastases are bones, lungs, and liver ( 12 ). Although there is no consensus about long-term postoperative follow-up, these patients should be monitored for recurrence ( 13 ).

During preoperative workup for neuroendocrine disorders such as paragangliomas, which may have adrenal or extra-adrenal localizations, an accurate definition of the disease or disease extension, and the nature of the lesions, can be possible by means of whole-body PET/CT imaging due to the simultaneous assessment of functional and anatomical information and the obtaining of standard uptake values of the lesions ( 14 , 15 ). It may be challenging to make differential diagnosis between the benign and malignant pathologies in a case with bilateral increased FDG uptake of adrenal glands ( 16 ). However, in the current patient, final assessment was decided in accordance with benign hyperplasia for adrenal uptakes and as a malignant tumor with regional metastasis for the renal lesion due to the more intense FDG uptake in the left renal mass observed by MRI and CT with its higher density (> 10 HU), and the presence of increased FDG uptake in the adjacent lymph node involvement. Another recommended functional imaging modality is ^123^I or ^131^I-metaiodobenzylguanidine (MIBG) scintigraphy, which has been used widely for the assessment of patients with paragangliomas. ^18^F-labelled fluoro-deoxy-glucose (^18^F-FDG) PET and somatostatin analogues labelled with gallium-68 may be used for detection of small lesions and metastatic lesions ( 17 ).

The primary aim of the treatment of malignant paragangliomas is surgical removal of the primary tumor and, if possible, the resection of the metastatic foci. In our patient, after complete surgical removal of the tumor and the metastatic lymph node, clinical and biochemical improvement was found. For inoperable tumors, radioactive isotop treatment with ^131^I-MIBG may provide symptomatic relief and some tumor shrinkage.

Only a few cases of paragangliomas as the cause of ectopic ACTH syndrome have been reported. To our knowledge, this is the first case of metastatic renal paraganglioma resulting in Cushing’s syndrome due to ectopic ACTH hypersecretion. However, we could not perform a genetic analysis of the patient using succinate dehidrogenase enzyme, particularly for SDHB mutations.
